# Cholinesterase inhibitors as Alzheimer's therapeutics

**DOI:** 10.3892/mmr.2019.10374

**Published:** 2019-06-11

**Authors:** Kamlesh Sharma

**Affiliations:** Department of Chemistry, Faculty of Physical Sciences, Shree Guru Gobind Singh Tricentenary University, Gurugram, Haryana 122505, India

**Keywords:** Alzheimer's, dementia, cholinesterase, inhibitor, multi-target

## Abstract

Alzheimer's disease (AD) is one of the most common forms of dementia. AD is a chronic syndrome of the central nervous system that causes a decline in cognitive function and language ability. Cholinergic deficiency is associated with AD, and various cholinesterase inhibitors have been developed for the treatment of AD, including naturally-derived inhibitors, synthetic analogues and hybrids. Currently, the available drugs for AD are predominantly cholinesterase inhibitors. However, the efficacy of these drugs is limited as they may cause adverse side effects and are not able to completely arrest the progression of the disease. Since AD is multifactorial disease, dual and multi-target inhibitors have been developed. The clinical applications and the limitations of the inhibitors used to treat AD are discussed in the present review. Additionally, this review presents the current status and future directions for the development of novel drugs with reduced toxicity and preserved pharmacological activity.

## 1. Introduction

Alzheimer's disease (AD) accounts for 60–70% of cases of dementia worldwide, with an estimated global incidence of 24.3 million cases. AD is a chronic syndrome that causes progressive deterioration of the central nervous system (CNS). AD causes progressive deficits in decision making, language, memory, learning, orientation and judgement (1). The major risk factor for AD is aging (2). However, physical exercise can decrease the rate of dementia (3).

The enzyme cholinesterase (ChE) is a significant therapeutic target for AD (4). The deterioration of cholinergic neurons in the brain and the loss of neurotransmission are the major causes of the decline in cognitive function in patients with AD (5).

According to the cholinergic hypothesis (5), the main cause of AD is the reduction in acetylcholine (ACh) synthesis. Therefore, one of the potential therapeutic strategies is to increase the cholinergic levels in the brain by inhibiting the biological activity of acetylcholinesterase (AChE). Therefore, AChE inhibitors are used to limit the degradation of ACh. AChE inhibitors are able to increase the function of neural cells by increasing the concentration of ACh (6).

The progressive synthesis and aggregation of β-amyloid (Aβ), a proteolytic fragment derived from amyloid precursor protein (APP), are additional critical factors involved in AD pathogenesis (7). Therefore, tacrine hybrids (8–10) and donepezil-based (11) dual inhibitors have been developed to inhibit both AchE activity and Aβ aggregation. Computational approaches have been used to design various dual inhibitors of AChE and Aβ cleaving enzyme 1 (12).

In addition, tauopathy is an important aspect of AD pathology, and τ protein hyperphosphorylation leads to the formation of intracellular neurofibrillary tangles of the microtubule-associated protein τ and subsequent neurodegeneration (13,14). Therapies targeting τ protein reduce and prevent its hyperphosphorylation and aggregation (15–17). Several drugs under development are in phase III clinical trials, including methylthioninium, which inhibits τ phosphorylation by activating the τ phosphatases or by inhibiting τ kinases (18,19).

Since AD is a multifactorial disorder, researchers have turned their attention to developing multi-target drugs to inhibit multiple factors involved in AD, including protein misfolding and associated Aβ aggregation, τ aggregation, metal dyshomeostasis, oxidative stress and the decreased ACh levels. However, few studies have been done to identify multi-target AD drugs (20,21).

## 2. Acetylcholinesterase

**Figure 1.**
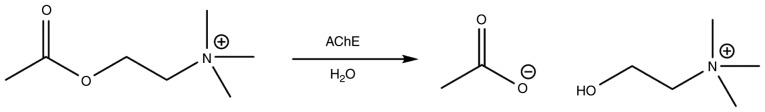
Schematic representation of AChE catalysis. AChE, acetylcholinesterase.

AChE (EC 3.1.1.7) (22) is an important enzyme involved in the cholinergic nervous system, which includes the peripheral nervous system and the CNS. AChE catalyses the hydrolysis of ACh to generate choline and acetate ions (Fig. 1). The active site of AChE is a large hydrophobic cavity. AChE consists of two subsites: i) The esteratic subsite (ES); and ii) the anionic substrate binding site (AS). ACh is a widely distributed neurotransmitter in the CNS. The AS binds to the positively charged quaternary amine of ACh, and can bind to other cationic substrates and inhibitors (22,23). The ES contains a catalytic triad consisting of Ser200, Glu327 and His440 (24). The catalytic triad is located ~20 Å from the enzyme surface, at the bottom of a narrow gorge that widens towards the base. As a part of the catalytic triad, Ser200 is responsible for the hydrolysis of choline esters by proton transfer (22,23). Additionally, the cation-π interaction is present between an aromatic amino acid and the quaternary ammonium of ACh (22).

The peculiar structural feature of the active site in the *Torpedo californica* AChE (TcAChE), a prototypical ACh-binding protein, consists of the presence of a high number of aromatic residues (~14 amino acids) (25). Trp84 is the most important aromatic amino acid for the AChE-ACh interaction, and its substitution with alanine results in a 3,000-fold decrease in reactivity (26). In addition to these sites, AChE possesses an ‘acyl pocket’, which confers substrate-specificity, and an ‘oxyanion hole’, which interacts with negative oxygen ions during catalysis, and increases the catalytic efficiency of AChE (27).

## 3. Traditional ChE inhibitors

A number of ChE inhibitors have been developed (28,29). Donepezil, galantamine, rivastigmine and memantine are the four drugs used to treat AD currently available on the market (30–32). However, the efficacy of these drugs is limited, and these drugs have shown various dose-associated side-effects, particularly at higher doses (28,29). Galantamine and donepezil are AChE inhibitors (28), whereas rivastigmine is a reversible inhibitor of both AChE and butyrylcholinesterase (BChE). Notably, donepezil is highly selective for AChE compared with BChE. The AChE inhibitory potencies (IC_50_ values) of tacrine, donepezil, rivastigmine and physostigmine are 77, 6.7, 4.3 and 0.67 nM, respectively (29).

### 

#### Physostigmine

**Figure 2.**
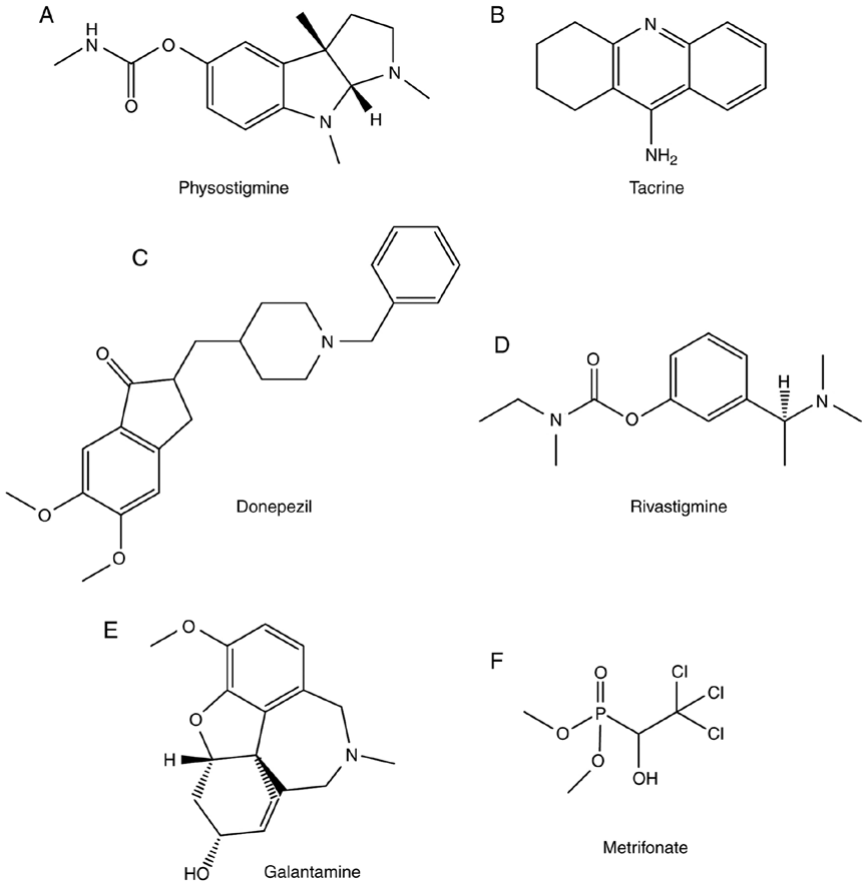
Traditional cholinesterase inhibitors. The molecular structures of (A) physostigmine, (B) tacrine, (C) donepezil, (D) rivastigmine, (E) galantamine and (F) metrifonate are presented.

Eserine, also known as physostigmine, was first isolated from Calabar beans in 1864 (33) and is an AChE inhibitor (34). Although physostigmine can cross the blood-brain barrier (BBB), this drug has a narrow therapeutic index due to its short half-life and numerous side effects (35). Its common side effects include diarrhoea, stomach cramps, increased production of saliva and excessive sweating (35). Due to these disadvantages, physostigmine was not approved for the treatment of AD. The structure of physostigmine is presented in Fig. 2A.

#### Tacrine

Tacrine was first synthesized in the 1930s, and was originally used as a muscle relaxant antagonist and respiratory stimulant (36). Tacrine has been used in patients with AD sincethe 1980s, having been approved by the FDA in 1993 and discontinued in 2013. The molecular structure of the drug is presented in Fig. 2B. Tacrine interacts with the amino acid residues Phe330 and Trp84, which are present in the ‘anionic site’ of AChE (37). Tacrine is an effective inhibitor of both AChE and BChE (38). However, the use of tacrine is limited due to its many side effects, including nausea, vomiting, loss of appetite, diarrhoea and clumsiness (39). In addition, patients treated with tacrine require blood monitoring due to the hepatotoxicity induced by this drug. Additionally, multiple-dosage regimens are required to maintain prolonged therapeutic activity, due to the short half-life of tacrine and its adverse side effects at high dosage (40). Tacrine was discontinued due to the aforementioned side effects and liver toxicity.

#### Donepezil

In 1996, the drug donepezil was approved for the treatment of mild to moderate AD (30) (Fig. 2C). However, donepezil presents various side effects, including insomnia, nausea, loss of appetite, diarrhoea, muscle cramps and muscle weakness (41). Patients treated with high doses of donepezil suffer from low blood pressure, severe vomiting, muscle weakness, severe nausea, breathing problems and bradycardia (41). In addition to inhibit ChE, donepezil may have additional mechanisms of action (42). Donepezil not only acts at the neurotransmitter level, but also at the molecular and cellular level in almost all stages involved in the pathogenesis of AD, including the inhibition of various aspects of glutamate-induced excitotoxicity, the reduction of early expression of inflammatory cytokines, the induction of a neuroprotective isoform of AChE and the reduction of oxidative stress-induced effects (42). Donepezil exhibits a unique molecular structure that causes the simultaneous inhibition of the active and the peripheral anionic sites (PAS) of TcAChE (43). However, donepezil does not directly interact with the oxyanion hole or the catalytic triad (43).

#### Rivastigmine

Rivastigmine was approved for the treatment of mild to moderate AD in 2000. In addition, this drug has been used for the treatment of Parkinson's disease-associated dementia (44). Although the exact mechanism of action of rivastigmine is unclear, it was hypothesized that it may exert its pharmacological action by increasing cholinergic function (32). Rivastigmine tartrate targets both BChE and AChE. Rivastigmine tartrate is a carbamate that binds to AChE, which cleaves rivastigmine into various phenolic derivatives that are rapidly excreted from the body (45). The carbamate moiety binds to the ES of AChE with more affinity than that of the acetate moiety of ACh during ACh hydrolysis. Therefore, the enzyme is inactivated for a certain amount of time (45). This effect may explain its unusually slow activation kinetics (32). Rivastigmine has major side effects, including stomach pain, weight loss, diarrhoea, loss of appetite, nausea and vomiting (46). An overdose of rivastigmine may cause numerous symptoms, including irregular, fast or slow breathing, chest pain, and slow or irregular heartbeat (46). The structure of rivastigmine is presented in Fig. 2D.

#### Galantamine

Galantamine is an alkaloid present in many plants, including daffodil bulbs (47). Galantamine has been used as a medicine in Russia and Eastern European countries for decades for the treatment of myopathy, myasthenia, and sensory and motor deficits associated with the CNS (48). Galantamine has also been shown to bind to nicotinic cholinergic receptors. Its activity against ChE was identified in the 1950s; it has been marketed with the name Nivalin and used for the treatment of several neurological diseases (49). Galantamine was approved for the treatment of AD in 2001 (31). The chemical structure of galantamine is presented in Fig. 2E. Galantamine has been shown to be effective in treating the cognitive symptoms of AD. Notably, a gradual increase in galantamine dosage may increase the tolerability of this drug (50). The main side effects of galantamine include convulsions, severe nausea, stomach cramps, vomiting, irregular breathing, confusion, muscle weakness and watering eyes (51).

#### Metrifonate

Metrifonate (Fig. 2F) is a long-acting organophosphate AChE inhibitor, and it is used for the treatment of schistosomiasis (52). Metrifonate can improve cholinergic neurotransmission via a pharmacologically active metabolite, 2,2-dichlorovinyl dimethyl phosphate, and has been tested for the treatment of AD (53). Metrifonate administered once per day can improve the cognitive function of patients with mild to moderate AD (53). The tolerability of metrifonate is good, but its long-term use cause adverse side effects, including problems with neuromuscular transmission and respiratory paralysis (25). Therefore, the development of this drug was interrupted during Phase III clinical trials.

## 4. Next-generation ChE inhibitors

Physostigmine derivatives, such as phenserine, tolserine and eseroline, have been developed as ChE inhibitors.

### 

#### Phenserine

Phenserine is a selective, non-competitive AChE inhibitor that not only inhibits AChE, but also reduces the production of APP *in vitro* and *in vivo* (54). Additionally, the toxicity of phenserine is lower compared with that of tacrine and physostigmine (55). Notably, treatment with phenserine was shown to improve memory and learning in aged dogs and rats (54). Phenserine was clinically tested for AD, but has shown only moderate success in initial Phase II clinical trials (54).

**Figure 3.**
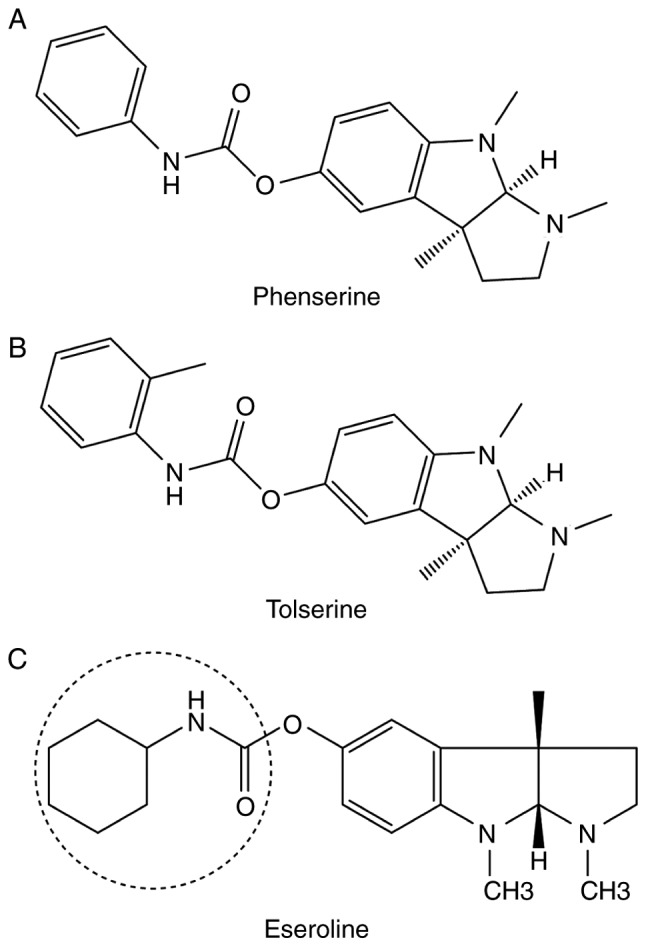
Novel cholinesterase inhibitors. The molecular structures of (A) phenserine, (B) tolserine and (C) eseroline are presented. The circle indicates the active moiety of eseroline.

Phenserine was observed to be a promising agent for the development of novel strategies for the treatment of AD due to its dual anti-Aβ and anti-AChE effects. However, in 2005, the biopharmaceutical company Axonyx, Inc. announced that phenserine was ineffective in two curtailed Phase III clinical trials (56). Furthermore, in 2010, a previous study demonstrated that high doses of phenserine may improve the symptoms of patients with mild to moderate AD (57). In 2016, it was demonstrated that phenserine also exhibits non-cholinergic effects with clinical potential. Phenserine was used for the treatment of cognitive impairments induced by traumatic brain injury in mice (58). Notably, clinical trials and the investigation of its mechanisms are currently under development (59). The structure of phenserine is presented in Fig. 3A.

#### Tolserine

The structure of tolserine slightly differs from that of phenserine by the presence of a 2-methyl group in its phenylcarbamoyl moiety (Fig. 3B). In 2000, preclinical studies concluded that tolserine is 200-fold more selective against human AChE (hAChE) compared with BChE. The inhibitory concentration of tolserine against AChE in human erythrocytes is 0.01 µM (60). Furthermore, its inhibitory concentration against human AChE in red blood cells pre-treated for 30 min using the Ellman technique is 0.0103 µM (61). The potency of tolserine against hAChE is higher compared with that of phenserine or physostigmine (62). However, its side effects or benefits in clinical and preclinical models are unclear.

#### Eseroline

Eseroline acts as an opioid agonist (63). In 1982, it was demonstrated that eseroline is a metabolite of physostigmine; however, in contrast to physostigmine, the effect of eseroline on AChE inhibition is limited and reversible (64). Various physostigmine analogues have been analysed for ChE inhibition (65). A cyclic alkyl carbamate derived from eseroline (Fig. 3C) was found to be effective against AChE with high selectivity compared with BChE (65). However, to the best of the author's knowledge, no recent studies have reported on the effects of eseroline.

## 5. Naturally-derived inhibitors

### 

#### Huperzine (Hup)

**Figure 4.**
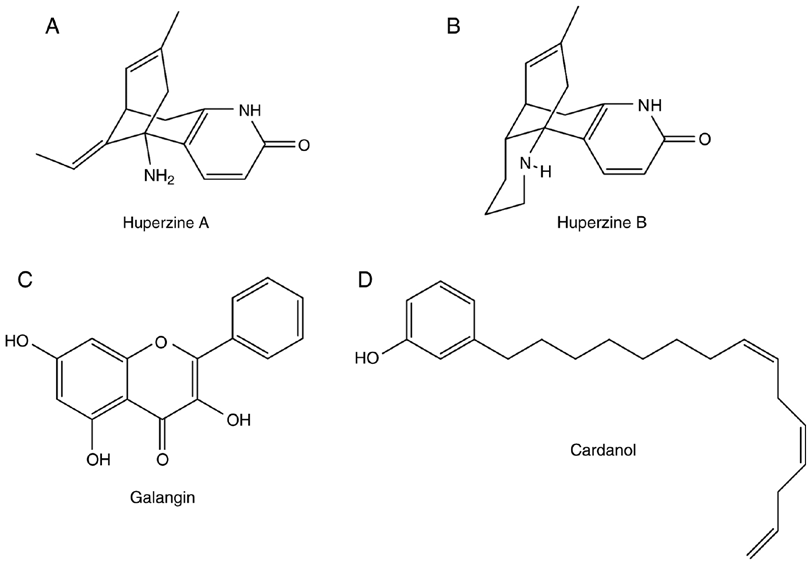
Naturally-derived cholinesterase inhibitors. The molecular structures of (A) huperzine A, (B) huperzine B, (C) galangin and (D) cardanol are presented.

Hup is a lycopodium alkaloid. Hup can be extracted and isolated from the herb *Huperzia serrata* (66). In total, two types of Hup are present: Hup-A and Hup-B (Fig. 4A and B, respectively). Hup-B is a natural homologue of Hup-A, which is used for the treatment of AD and age-related memory impairment, and for memory and learning enhancement, as it increases the level of ACh (67). Hup-A is more effective than rivastigmine, galantamine and tacrine (67). Hup-A is a highly selective and potent inhibitor of AChE. However, it is less active against BChE compared with AChE. Tacrine-Hup-A hybrids have shown potential AChE-inhibiting effects (67).

A prodrug of Hup-A called ZT-1 is under development for the treatment of AD. Both Hup-A and -B interact in similar ways with AChE (68). Both Hup molecules interact with anionic sites via π-π stacking, and with Trp84 and Phe330 via CH/π-interactions or van der Waals forces (68). The α-pyridone moiety of Hup interacts with the active site of AChE via CH/π-interactions and H-bonds. The carbonyl oxygen of Hup repels the carbonyl oxygen of Gly117. As a result, the peptide bond between Gly118 and Gly117 flips (68). Furthermore, the flipped peptide plane conformation is stabilized by H-bonds between the oxygen of Gly117 with the nitrogen atoms of Ala201 and Gly119 (68). However, Hup-A may cause mild cholinergic side effects such as nausea, vomiting and diarrhoea (69).

#### Flavonoid

Flavonoids have attracted great interest due to their free-radical-scavenging properties. A series of flavonoid compounds have shown effective AChE inhibitory activities *in vitro* (70). Galangin, a flavonol derived from the rhizomes of *Alpiniae officinarum*, has shown potent inhibitory activity against AChE (Fig. 4C) (70). However, the toxicity of these flavonoids have not been investigated in preclinical and clinical trials, and no human trials have been reported.

#### Cardanol

In 2009, various non-isoprenoid phenolic lipids obtained from *Anacardium occidentale* were investigated for their inhibitory activity against AChE (71). In particular, cardanol, a phenolic lipid, has shown promising results (71). Moreover, cardanol can be extracted from cashew nut shells (72). However, its toxicity has not yet been investigated in preclinical and clinical trials. The molecular structure of cardanol is presented in Fig. 4D.

## 6. Hybrid inhibitors

### 

#### Donepezil-AP2238 hybrid

**Figure 5.**
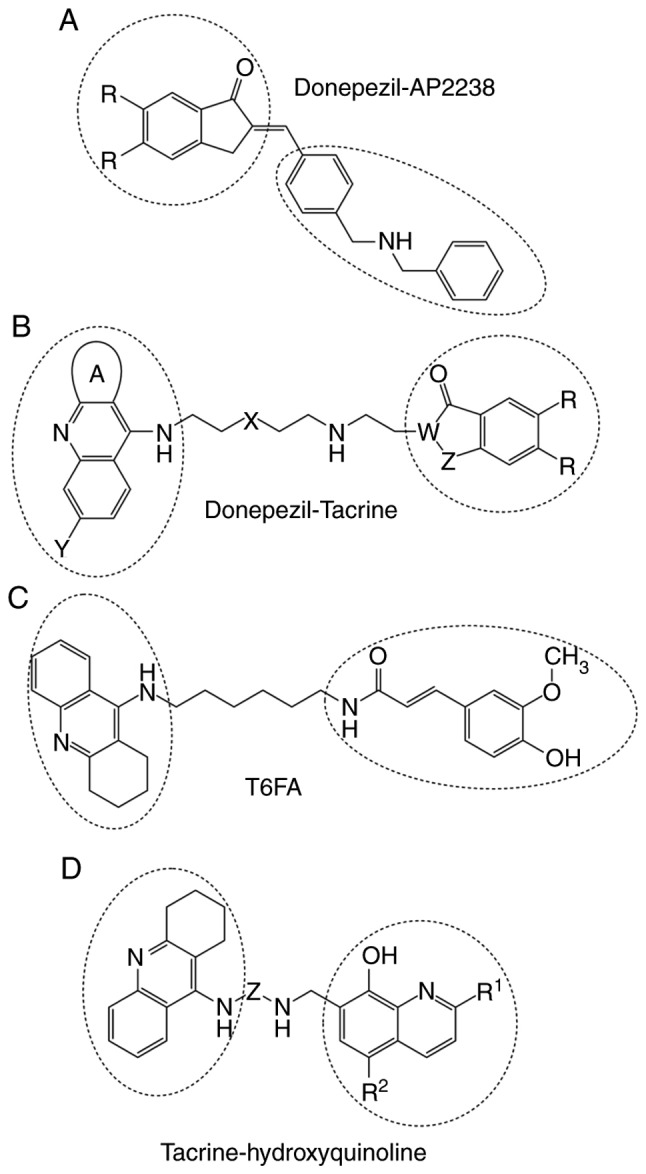
Hybrid cholinesterase inhibitors. The molecular structures of (A) donepezil-AP2238, (B) donepezil-tacrine, (C) T6FA and (D) tacrine-hydroxyquinoline are presented. The drugs forming the hybrids are indicated by circles.

AP2238 was the first developed drug with dual binding sites, and it is able to interact with both anionic sites of AChE (73). The activities of AP2238 and donepezil against AChE are similar. However, the effect of AP2238 in inhibiting Aβ-mediated toxicity is higher (73). Therefore, a series of donepezil-AP2238 hybrids have been investigated (74). The structure of a donepezil-AP2238 hybrid is presented in Fig. 5A. Out of 22 compounds investigated (74), two molecules have shown potent activities. Both compounds have an alkyl chain of five carbon atoms and an amino group present at the end of the chain, which results in an increased interaction with the PAS of AChE (74).

#### Donepezil-tacrine hybrid

Camps *et al* (11) designed a series of donepezil-tacrine hybrids (Fig. 5B), which interact simultaneously with the active, peripheral and mid-gorge binding sites of AChE. These hybrids were found to inhibit AChE, BChE and Aβ-aggregation induced by AChE. Donepezil-tacrine hybrids are synthesized by combining 6-chlorotacrine with the indanone moiety of donepezil, and are more effective at inhibiting hAChE compared with their parent compounds (11).

#### Tacrine-ferulic acid (T6FA) hybrid

T6FA hybrid has shown more potent AChE-inhibitory effects compared with tacrine, and inhibits BChE at comparable levels (Fig. 5C). T6FA has shown potent activity in inhibiting Aβ-mediated AD-associated pathogenesis *in vitro* and *in vivo* (75).

#### Tacrine and 8-hydroxyquinoline hybrids

Tacrine and 8-hydroxyquinoline hybrids are drugs that inhibit cholinesterase and reduce Aβ aggregation by forming complexes with redox-active metals (Fig. 5D). These hybrids inhibit AChE more effectively than tacrine alone, and have been shown to have increased CNS permeability, low toxicity, and antioxidant and copper complexing properties (38).

L-monoamine oxidases (MAOs) (EC 1.4.3.4) catalyse the oxidation of monoamines (76,77). Recently, a donepezil-chromone-melatonin hybrid has been developed as a multi-target agent with strong BChE and moderate hAChE inhibitory capacities, and with anti-MAO-A/B and antioxidant properties (78). Furthermore, tacrine-acridine hybrids have been developed as multi-target drugs for the treatment of AD (79). In addition, tacrine-carbohydrate (80) and tacrin-T6FA (81) hybrids have shown potent ChE inhibitory potential.

## 7. Synthetic analogues

Synthetic analogues have been developed as competitive ChE inhibitors, since gastrointestinal side effects and hepatotoxicity can be avoided with targeted pharmacological development (82). However, the main problem of synthetic analogues is that they may not permeate the BBB and their effectiveness can be lower compared with naturally derived ChE inhibitors (83).

### 

#### Tacrine analogues

**Figure 6.**
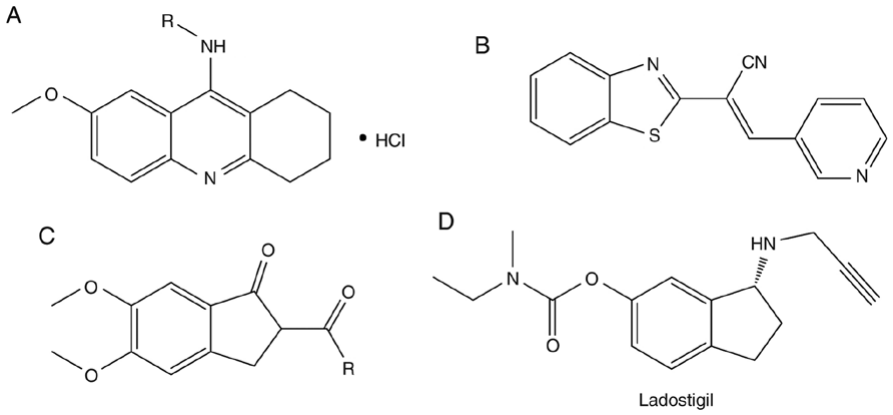
Synthetic analogues of cholinesterase inhibitors. (A) Tacrine analogue, (B) heteroarylacrylonitrile derivative, (C) indenyl derivative and (D) Ladostigil.

N-alkyl-7-methoxytacrine hydrochloride (Fig. 6A), an analogue of tacrine, has shown improved AChE-inhibitory activities compared with the parent drug tacrine (84).

#### (E)-2(benzo[d]thiazol-2-yl)-3-heteroarylacrylonitriles

(E)-2(Benzo[d]thiazol-2-yl)-3-heteroarylacrylonitriles have been in development as AChE inhibitors since 2012 (Fig. 6B) (84). The most potent compound among them was found to be more selective to AChE than galanthamine.

#### Indenyl derivatives

Various analogues of phenyl-5,6-dimethoxy-1-oxo-2,3-dihydro-1H-2-indenylmethanone were synthesized and tested by Ali *et al* in 2009 (83). Most of them showed moderate AChE-inhibitory effects. Ali *et al* (83) suggested that the presence of methoxy groups on the phenyl ring significantly improved the inhibition of AChE (Fig. 6C).

#### Ladostigil

Ladostigil is a potent anti-AD drug with AChE-inhibitory and neuroprotective properties (Fig. 6D). Ladostigil [(N-propargyl-(3R) aminoindan-5yl)-ethyl methyl carbamate)] is in Phase IIb trials (85).

Recently, 1,2,4-triazine scaffolds (86) and 1,2,3-triazole-chromenone carboxamide derivatives (87) have been developed as multi-target therapeutic agents for the treatment of AD. Chalcone-based derivatives have shown ChE-inhibitory properties (88). Chromone scaffolds have shown dual inhibition of ChE and MAO (89). Various donepezil-based multi-functional ChE inhibitors have been developed for the treatment of AD (90).

## 8. Future directions

Since the discovery of the first AchE inhibitor, physostigmine (30), a large number of studies have been performed to identify more effective inhibitors. Traditional inhibitors are naturally-derived agents. Other inhibitors include analogues of the traditional inhibitors, derivatives of natural compounds and hybrids of synthetic inhibitors. These inhibitors cause milder side effects than traditional drugs and may have improved properties, such as better BBB permeability and increased effectiveness (11,67). In addition, these compounds are able to limit the progression of AD. Recent reports investigated AChE inhibition (80,88,90), but only a few novel drugs have been tested in humans (18,60–62,78). Most of these inhibitors have been studied in animal models, or using *in vitro* and *in silico* models. Therefore, further studies in humans to investigate the safety, efficacy and toxicity of these drugs are required.

AChE inhibitors are not able to completely stop the progression of AD, and various single-target drugs that have reached clinical trials were not able to effectively treat AD. Therefore, there is a need to develop multi-functional drugs that are able to targ*et al*l symptoms of AD, including the decreased levels of ACh, protein misfolding and associated Aβ aggregation, hyperphosphorylation of τ protein, metal dyshomeostasis and oxidative stress. However, only a limited number of studies have focused on the development of multi-target drugs (79,81,89).

According to structure-activity relationship studies, the design of novel potent multi-target inhibitors should have the following characteristics: i) The presence of a nitrogen atom with a positive charge (91); ii) the size of the alkyl chain attached to the nitrogen atom should be small, such as a methyl group (92); iii) the presence of an oxygen atom able to form hydrogen bonds, such as an ester (93); iv) the presence of electron-donating groups such as hydroxyl and methoxy groups (83); and v) the presence of a two-carbon unit between nitrogen and oxygen atoms (91). Notably, the overall size of the molecule should be small, since large molecules can exhibit decreased activity (94).

## 9. Conclusions

The present review provided an overview of the ChE and AChE inhibitors that have been developed to treat AD. These inhibitors include naturally-derived inhibitors, synthetic analogues and hybrids. Although ChE inhibitors do not cure AD, these drugs are recommended to limit neurodegeneration in patients with AD. Since current ChE inhibitors can cause several side effects, the development of novel agents with different structures and mechanisms of action is required. Since AD is a multifactorial disease, multi-target inhibitors should be developed. Therefore, future approaches should be focused on the development of a single molecule able to target multiple factors involved in AD. To the best of the author's knowledge, only a limited number of studies have used this approach. The development of a multi-target drug is a challenging task that can be accomplished by using computational approaches, including molecular modelling and molecular docking (95). These methods can provide helpful insights into the design of novel inhibitors, reducing the time and costs of development. The present review may be helpful to medicinal chemists and to the pharmaceutical industry in designing and developing novel drugs for the treatment of AD.

## Data Availability

Not applicable.
